# Evaluating the prevalence and severity of NAFLD in primary care: the EPSONIP study protocol

**DOI:** 10.1186/s12876-021-01763-z

**Published:** 2021-04-20

**Authors:** Patrik Nasr, Fredrik Iredahl, Nils Dahlström, Karin Rådholm, Pontus Henriksson, Gunnar Cedersund, Olof Dahlqvist Leinhard, Tino Ebbers, Joakim Alfredsson, Carl-Johan Carlhäll, Peter Lundberg, Stergios Kechagias, Mattias Ekstedt

**Affiliations:** 1 Department of Health, Medicine and Caring Sciences, Linköping University, Linköping, Sweden; 2 Center for Medical Image Science and Visualization (CMIV), Linköping University, Linköping, Sweden; 3 Department of Biomedical Engineering, Linköping University, Linköping, Sweden; 4 AMRA Medical AB, Linköping, Sweden; 5 Department of Clinical Physiology in Linköping, Department of Health, Medicine and Caring Sciences, Linköping University, Linköping, Sweden

**Keywords:** Non-alcoholic fatty liver disease, Type 2 diabetes mellitus, T2DM, Cirrhosis, Biomarkers

## Abstract

**Background:**

Non-alcoholic fatty liver disease (NAFLD) affects 20–30% of the general adult population. NAFLD patients with type 2 diabetes mellitus (T2DM) are at an increased risk of advanced fibrosis, which puts them at risk of cardiovascular complications, hepatocellular carcinoma, or liver failure. Liver biopsy is the gold standard for assessing hepatic fibrosis. However, its utility is inherently limited. Consequently, the prevalence and characteristics of T2DM patients with advanced fibrosis are unknown. Therefore, the purpose of the current study is to evaluate the prevalence and severity of NAFLD in patients with T2DM by recruiting participants from primary care, using the latest imaging modalities, to collect a cohort of well phenotyped patients.

**Methods:**

We will prospectively recruit 400 patients with T2DM using biomarkers to assess their status. Specifically, we will evaluate liver fat content using magnetic resonance imaging (MRI); hepatic fibrosis using MR elastography and vibration-controlled transient elastography; muscle composition and body fat distribution using water-fat separated whole body MRI; and cardiac function, structure, and tissue characteristics, using cardiovascular MRI.

**Discussion:**

We expect that the study will uncover potential mechanisms of advanced hepatic fibrosis in NAFLD and T2DM and equip the clinician with better diagnostic tools for the care of T2DM patients with NAFLD.

*Trial registration:* Clinicaltrials.gov, identifier NCT03864510. Registered 6 March 2019, https://clinicaltrials.gov/ct2/show/NCT03864510.

**Supplementary Information:**

The online version contains supplementary material available at 10.1186/s12876-021-01763-z.

## Background

Non-alcoholic fatty liver disease (NAFLD) is the most common chronic liver disease, with a worldwide prevalence of 20–30% [1]. Histological features range from hepatic steatosis to non-alcoholic steatohepatitis (NASH), the latter being characterized by inflammation, with or without fibrosis, with the risk of progressing to cirrhosis [2]. Cirrhosis, in turn, is associated with a 2.5% annual risk of developing hepatocellular carcinoma (HCC) [3]. In the near future, NAFLD is expected to become the leading cause for liver transplantation [4].

NAFLD increases the risk of liver-related and cardiovascular morbidity and mortality [5, 6]. Clinical and histological variables that predict overall mortality in NAFLD are age, type 2 diabetes mellitus (T2DM) [7, 8], and liver fibrosis [7, 9, 10].

In the last four decades, there has been a steep increase in T2DM global prevalence, both in high/middle-, and low-income countries [11]. And although NAFLD is strongly associated with the metabolic syndrome and T2DM [12], the association is bidirectional; with a markedly higher prevalence of NAFLD in patients with T2DM (40–70%) than in individuals without T2DM, and an increased incidence of T2DM in patients with NAFLD [13, 14].

Liver biopsy is the current gold standard for diagnosing severity of NAFLD. However, it has several limitations, including adverse events as well as sampling and observer variability [15–17]. Consequently, non-invasive methods are being evaluated to replace liver biopsy. These include magnetic resonance (MR)-based methods and serological marker testing.

Recently, the European Association for the Study of Diabetes (EASD), Obesity (EASO) and the Liver (EASL) proposed non-invasive screening for NAFLD and advanced fibrosis among patients with T2DM [18]. Nevertheless, to date, there is no clear consensus on how to implement these guidelines. Furthermore, concerns have been raised on whether screening is cost-effective, especially since most available non-invasive tests have low positive predictive value and because treatment of liver fibrosis is lacking [19].

Hepatic fat can be assessed directly by proton density fat fraction (PDFF) using magnetic resonance techniques [20, 21]. However, hepatic fibrosis has no molecular signature that can be detected and is therefore assessed indirectly by quantification of liver “stiffness” (or “elasticity”) [20]. The most accurate non-invasive methods for assessing stiffness include transient elastography (TE) and MR elastography (MRE).

Serological markers for the evaluation of liver fibrosis are more accessible and easier to use than imaging and therefore preferable for the evaluation of a prevalent disease. However, no panel of serological fibrosis markers has shown clinically acceptable sensitivity required for the diagnosis of advanced fibrosis although they can be used to exclude advanced fibrosis [24].

Most NAFLD-studies in patients with T2DM have been performed at tertiary centers where patients are more likely to have advanced NAFLD. In Sweden, most patients with T2DM is cared for in primary care. Hence, hospital series of NAFLD in T2DM patients is misleading. Therefore, we aim to recruit study participants from primary care, using the latest imaging techniques, to collect a cohort of well phenotyped patients with T2DM to evaluate the prevalence and severity of NAFLD in primary care.

## Methods/design

### Overview

**Fig. 1:**
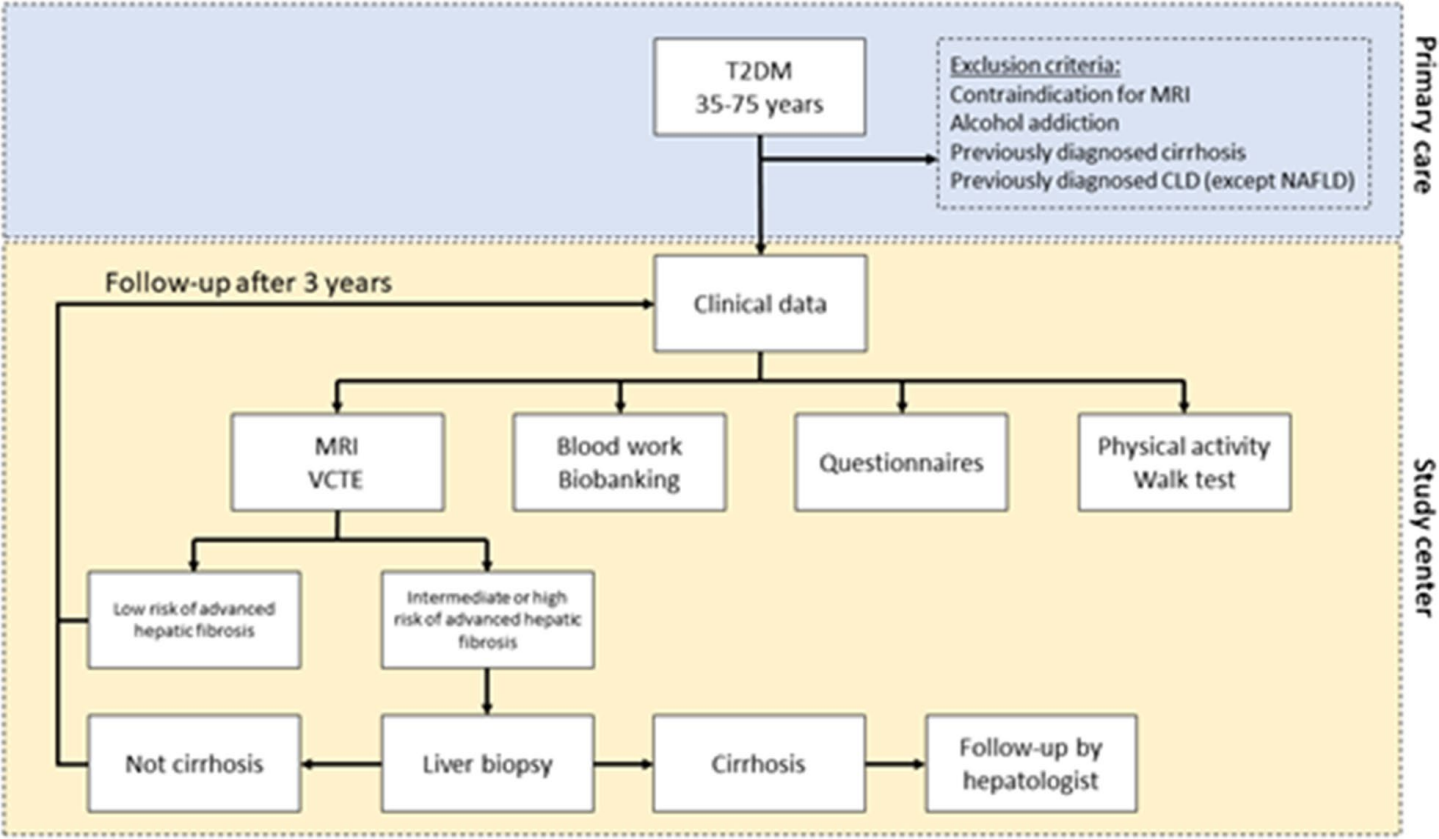
Study flow chart outlining patient recruitment, inclusion, data collection and follow-up. CLD, chronic liver disease; MRI, magnetic resonance imaging; NAFLD, non-alcoholic fatty liver disease; T2DM, type 2 diabetes mellitus; VCTE, vibration controlled transient elastography

The EPSONIP (**E**valuating the **P**revalence and **S**everity **O**f **N**AFLD in **P**rimary Care) study is a prospective cohort study with the aim to recruit 400 highly phenotyped patients with T2DM from primary care that will facilitate cross-sectional as well as longitudinal and long-term analyses. The comprehensive study protocol includes clinical information, fitness assessment, physical activity in everyday life, magnetic resonance imaging, lifestyle, and quality of life (QoL) data, as well as a biological sample collection (including genetic analysis). Specifically, we will evaluate liver fat content using magnetic resonance imaging (MRI); hepatic fibrosis, using magnetic resonance elastography (MRE) and vibration-controlled transient elastography (VCTE); fat free muscle volume, muscle fat infiltration, and abdominal fat distribution, using whole body fat–water separated MRI; and cardiac function and structure, using cardiovascular MRI (cardiovascular MRI will be assessed in 200 participants). Each participant will be investigated twice, at 3-year intervals, to identify individuals that develop cardiovascular disease and comorbidities, as well as progressive liver disease. See Fig. 1 for an overview of study procedures.

### Objectives

The overall purpose of the study is to better characterize the T2DM-NAFLD patient population using non-invasive methods to inform the diagnosis and care for this population. We aim to do this by identifying the relationship between T2DM, NAFLD, advanced hepatic fibrosis and myocardial dysfunction, as well as clinical, biochemical, and lifestyle parameters of patients with T2DM and NAFLD.

#### Specific aims

1. To determine the prevalence and incidence of NAFLD and advanced fibrosis in participants with T2DM using advanced non-invasive approaches.
2. To test whether serological markers enable a faster and better-informed assessment than liver biopsy of the risk of advanced hepatic fibrosis, myocardial fibrosis, or myocardial dysfunction in participants with T2DM.
3. To identify the clinical, biochemical, putative pathophysiological, and genetic factors associated with NAFLD and advanced fibrosis in participants with T2DM
4. To determine the relationship between NAFLD, myocardial function, and clinical, biochemical, and lifestyle factors in patients with and without T2DM.
5. To identify non-invasive biomarkers that predict fibrosis progression and altered myocardial function in NAFLD patients with and without T2DM.

### Organization and oversight

The study is run and coordinated by Dr. Mattias Ekstedt (PI) at the Department of Gastroenterology and Hepatology, University Hospital in Linköping, and Faculty of Medicine and Health Sciences, Linköping University. The study will be monitored by Forum Östergötland.

Patients will be recruited at primary healthcare centers. At present, four healthcare centers in South East Sweden (in Östergötland) are part of the study (Ekholmen and Kärna in Linköping, and Åby, Helsa Vårdcentral Kneippen and Cityhälsan Söder in Norrköping). Patient recruitment at each health care center will be overseen by General Practitioners participating in the study. The PI will supervise activities during regular on-site meetings. Collection of clinical data and blood samples will be performed by experienced research nurses. MRI will be performed at Center for Medical Image Science and Visualization (CMIV) in Linköping and the Department of Radiology at Vrinnevisjukhuset in Norrköping.

### Ethics approval and consent to participate

All recruitment and attaining written informed consent are conducted according to nationally accepted practice and in full accordance with the World Medical Association of Helsinki 2018. Data is collected and processed in accordance with the applicable General Data Protection Regulation (EU) 2016/679 (GDPR) legislation, and in compliance with the International Conference of Harmonization—Good Clinical Practice (ICH-GCP) requirements [22].

The EPSONIP study was approved by the Regional Ethical Board of Östergötland 2018/176-31 and 2018/494-32 and is registered as a clinical trial (clinicaltrials.gov identifier NCT 03864510). A complementary amendment, titled EPSONIP—Sleep, has been approved by the Swedish Ethical Review Authority 2019-03854.

### Participants

Patients with T2DM attending annual check-ups at their primary healthcare center will be eligible for inclusion in the study. Patients will be invited to participate by their diabetes nurse or treating physician. Patients of both sexes will be consecutively included. The inclusion and exclusion criteria are presented in Table 1

**Table 1:** Inclusion and exclusion criteria

Criteria	
Inclusion	Diagnosis of T2DM according to current guidelines
	Age: 35–75 years
Exclusion	Contraindications to perform MRI (pacemaker, ferrous metal implants/fragments, claustrophobia, extreme obesity, and/or pregnancy)
	Alcohol dependence
	Previously diagnosed liver cirrhosis
	Previously diagnosed primary liver disease (except NAFLD)

Following receipt of information about the study and the opportunity to ask questions, participants will be asked to provide written informed consent, witnessed, and dated, by a member of the research team. Written informed consent will always be obtained prior to study-specific procedures.

### Estimation of sample size and power calculation

The cohort size was primarily defined so that a significant number of patients with significant or progressive fibrosis was identified. In previous studies of diabetic patients, 45–65% had fatty liver, and 7% had significant fibrosis [23]. Extrapolated to our study, that would give 260 patients with fatty liver and 28 patients with advanced fibrosis at baseline. At follow-up, one third of the patients with fatty liver is expected to progress in fibrosis stage. Therefore, we expect that approximately 86 patients will have a progressive disease state.

For the majority of our aims, no power calculation can be performed. But to ascertain that we had significant power to investigate non-invasive markers relevant for this patient cohort we made power calculations for one baseline and one follow-up parameter. Given the focus of the study on ectopic fat accumulation we wanted to ascertain that we had sufficient power to detect a difference in epicardial fat accumulation between diabetic patients with and without fatty liver at baseline. In a previous study performed by our group, patients with diabetes had an epicardial fat volume of 62.1 ± 21.0 mL/m^2^. We expect at least a 10% difference between groups. The power calculation indicated, with 80% power, that 90 patients would be sufficient to detect a difference at an α value of 0.05. For the follow-up assessment we decided to use end diastolic volume (EDV) as a surrogate for cardiac remodeling. In our patients with diabetes the EDV was 69.6 ± 15.2 mL/m^2^. We expect at least a 10% difference between groups. The power calculation indicated, with 80% power, that 146 patients would be sufficient to detect a difference at an α value of 0.05.

### Study procedures

Following the provision of informed consent, patients will be assigned a unique study-participate identification code incorporating the recruitment site identifier. All data will be link-anonymized throughout the study, recorded through a secure web-based application for electronic data (REDCap™).

Patients that have consented to participate in the study will participate in a subsequent study visit at one of two sites (Linköping or Norrköping). A member of the research team will complete a clinical report form on clinical data (Table 2), with special focus on T2DM history and treatment. Questionnaires regarding the lifestyle and self-reported quality of life will be obtained, fitness and physical activity will be assessed, and baseline clinical biochemistry will be obtained.

**Table 2:** Layout of the anthropometric and clinical data collected at inclusion and 3 year follow-up

Categories of data	
Basic data	
Date of birth	
Gender	
Anthropometrics	
Height (cm)	
Weight (kg)	
Waist circumference (cm)	
Hip circumference (cm)	
Blood pressure (mmHg)	
Medical history	
Date of T2DM diagnosis	
Current or recent medication (including over-the-counter, traditional/herbal remedies, and nutritional supplements)	
Relevant comorbidities and date of diagnosis, including	
Hypertension, dyslipidemia	
Ischemic heart disease, including PCI and CABG	
Congestive heart failure	
Stroke	
Malignancies	
Lifestyle	
Smoking—yes/no/ex, and frequency of smoking (pack-years)	
Coffee consumption—cups/days	
Alcohol consumption	
Physical activity and fitness	
Patient reported quality of life	
Sleep quality assessment	
Family history	
Family medical history	

#### Questionnaires

Four validated questionnaires will be used to assess lifestyle factors relevant to NAFLD:

1. Alcohol questionnaires
  AUDIT: The Alcohol Use Disorders Identification Test (AUDIT), a screening tool developed by the World Health Organization to assess alcohol consumption, drinking behaviors, and alcohol-related problems [24].
  LDH: Lifetime Drinking History, designed to provide quantitative indices of an individual’s alcohol consumption patterns from the onset of regular drinking [25].
2. Lifestyle questionnaire
  Dietary and physical activity variables are assessed using the validated questionnaires developed by the National Board of Health and Welfare in Sweden [26, 27].
  IFIS: The International Fitness Scale, to assess self-reported fitness [28].
  ESS: Epworth Sleepiness Scale [29].
  PSQI: Pittsburgh Sleep Quality Index [30].
  STOP-Bang: Obstructive sleep apnea questionnaire [31].
3. Patient-reported quality of life.
  EQ-5D-5L: This questionnaire was developed by the EuroQol Group in 2009 as a measure of health-related quality of life. The descriptive system comprises five dimensions: mobility, self-care, usual activities, pain/discomfort, and anxiety/depression [32].

#### Fitness and physical activity

Lifestyle and NAFLD are closely related. Accordingly, we will perform an objective cardiorespiratory fitness measurement (6-min walk test) and a 7 day registration of objective physical activity using an accelerometer (Actigraph^®^ GT3X, Pensacola, FL, USA).

- The 6-min walk test is a submaximal functional exercise test that measures the distance walked over a period of 6 min [33]. The 6-min walk distance provides a measure for integrated global response of multiple cardiopulmonary and musculoskeletal systems involved in exercise, i.e., cardiorespiratory fitness.
- Physical activity over 7 d will be recorded using Actigraph^®^ GT3X, a small non-invasive accelerometer worn on the wrist, that captures and records continuous, high-resolution physical activity and sleep/wake information.
- One overnight registration of signs related to obstructive sleep apnea recorded by home respiratory polygraphy.

#### Blood sample acquisition and biorepository

**Fig. 2:**
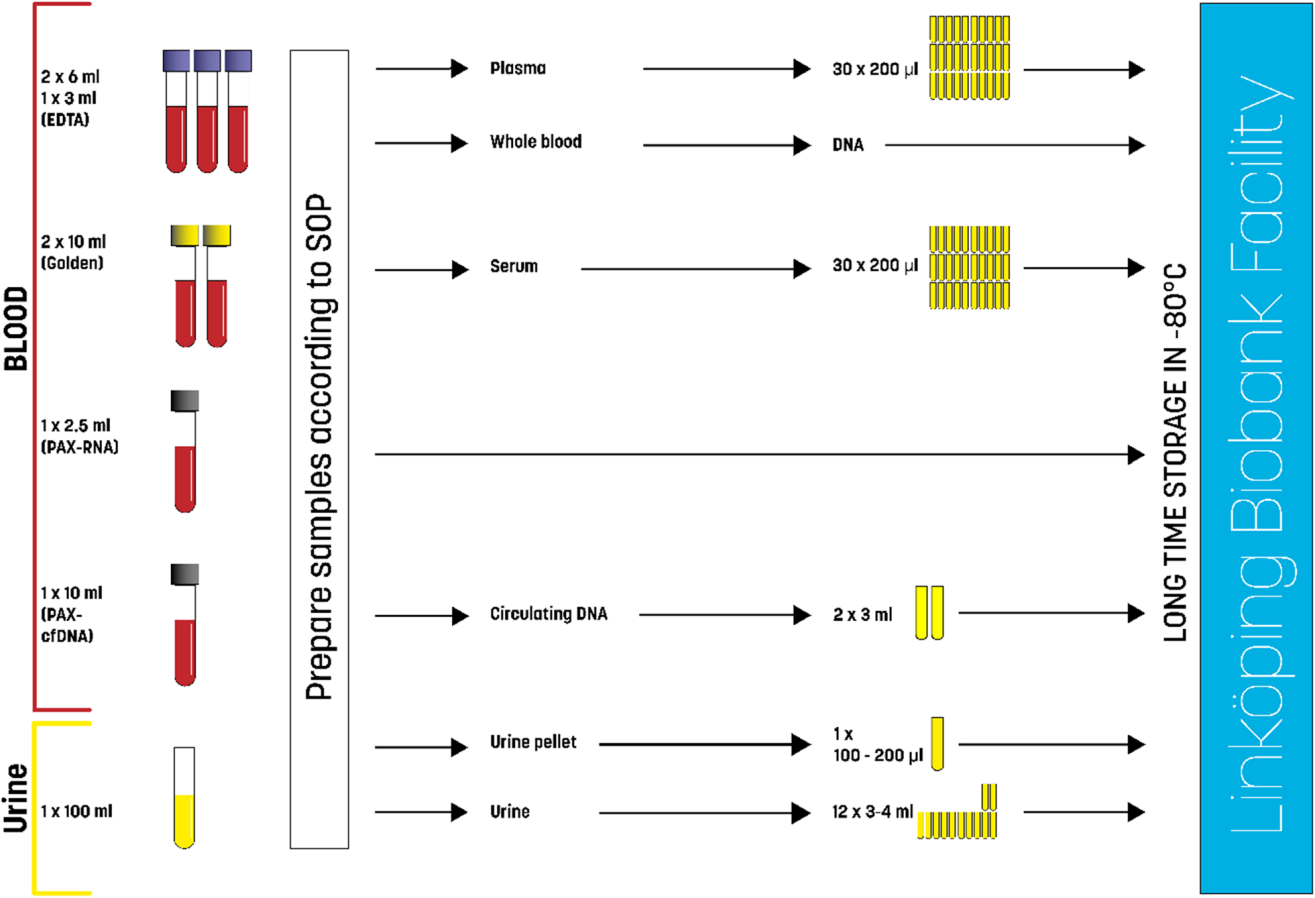
Overview of blood sample acquisition and biorepository

Each patient will be asked to provide blood and urine samples that will be physically stored at the Linköping Biobank Facility (Fig. 2). The biobank facility is a collaboration between the Faculty of Medicine and Health Sciences at Linköping University and Region Östergötland, with a state-of-the-art facility for quality-controlled storage in secure freezers. All samples are collected after an overnight fast. The following amounts of blood and urine will be collected from each patient:

- 3 × 5 mL, into EDTA Vacutainer tubes for **plasma** collection, for subsequent analyses of fibrosis biomarkers and metabolomics/proteomics/lipidomics
- 4 × 5 mL, into red top Vacutainer tubes for **serum** collection, for subsequent analyses of fibrosis biomarkers or metabolomics/proteomics/lipidomics
- 1 × 5 mL, into PAX tube for **RNA** preservation, for blood RNA extraction for transcriptomic analysis
- 1 × 10 mL, into PAX tube, for extraction of circulating cell-free **DNA**
- 1 × 100 mL urine into a sterile collecting vial, after centrifugation urine pellet is collected into a 2 ml cryovial and urine is stored in 12 × 4 ml cryovials

#### Clinical hematology/biochemistry/immunology/virology

Several blood, serum and plasma markers will be analyzed (Table 3) to exclude other causes of chronic liver disease, obtain a metabolic profile and to calculate previously identified biomarker algorithms associated with advanced fibrosis (Additional file 1: Table S1).

**Table 3:** Detailed blood/serum/plasma work-up

Blood/serum/plasma markers	
Complete blood count	
Hemoglobin, hematocrit, mean corpuscular value, mean corpuscular hemoglobin, mean corpuscular hemoglobin concentration, platelets, white blood cells	
Liver tests	
Albumin, bilirubin, ALT, AST, ALP, γGT, PT(INR), high sensitive C-reactive protein	
Iron studies	
Iron, transferrin saturation, total iron binding capacity, ferritin	
Serum protein electrophoresis	
Antitrypsin, albumin, orosomucoid, haptoglobin, immunoglobulins (IgG, IgM, IgA)	
Minor kidney function panel	
Sodium (Na), potassium (K), creatinine	
Metabolic tests	
Cholesterol, triglycerides, LDL, HDL. fasting glucose, C-peptide, HbA1c, insulin, GAD-antibody	
Direct alcohol marker	
Phosphatidylethanol	
Auto-antibody screen	
ANA, anti-LKM antibody, anti-SM antibody, anti-M antibody	
Viral serology	
HBsAg, anti-HBc, anti-HCV, Hep A IgM, Hep A IgG	

ALP, alkaline phosphatase; ALT, alanine aminotransferase; ANA, antinuclear antibody; AST, aspartate aminotransferase; GAD, glutamic acid decarboxylase; γGT, gamma-glutamyl transferase; HbA1c, hemoglobin A1c; HBC, hepatitis B core; HBsAg, hepatitis B surface antigen; HCV, hepatitis C virus; HDL, high-density lipoprotein; INR, international normalized ratio; LKM, liver-kidney microsomal; LDL, low-density lipoprotein; M, mitochondrial; PT, prothrombin time; SM, smooth muscle

### Multimodal MR examination

**Fig. 3:**
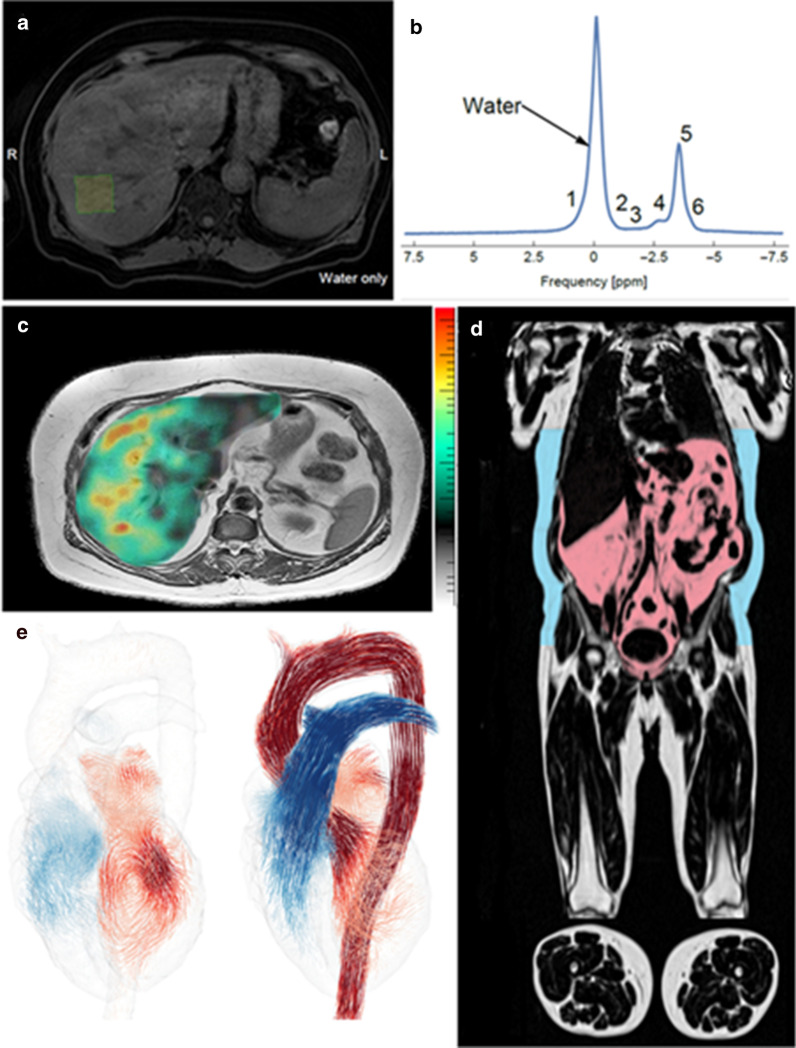
Image A shows the representative water MR image with the placement of a proton magnetic resonance spectroscopy (^1^H-MRS) voxel in the right hepatic lobe. Image B shows in vivo ^1^H-MRS spectrum for water and fat. Image C shows MRE for a cirrhotic NAFLD patient. Image E shows a whole-body water-fat separated imaging for quantification of visceral and subcutaneous adipose tissue volume. And image D shows a 4D flow image of a healthy heart. 4D flow, four-dimensional flow; ^1^H-MRS, proton magnetic resonance spectroscopy; MR, magnetic resonance; MRE, MR elastography

We have devised a multimodal MR-protocol for this project that includes a range of specific MR-techniques, including determination of iron concentrations in the liver, PDFF determination using MRS to measure hepatic triglyceride concentration (Fig. 3a, b), and 3D-MRE to determine the hepatic fibrosis stage (Fig. 3c). The cardiac investigations will include cine morphological MRI, a reference method for volumetric assessment, including left ventricular size, stroke volume and ejection fraction; native 3D-QALAS for 3D mapping of T1 and T2 relaxation time in the whole myocardium in one breath-hold, to estimate diffuse myocardial fibrosis; cardiac DIXON imaging, to determine epi- and paracardial adipose tissue; and 4D flow MRI, for the assessment of blood flow and model-based assessment of cardiac function (Fig. 3e). Furthermore, the protocol will include whole body water-fat separated imaging for quantification of visceral and subcutaneous adipose tissue volume, fat free thigh muscle volume, and muscle fat infiltration (Fig. 3d). The protocol will be used for data acquisition on 1.5 T MR-scanners (Philips Healthcare, Best, The Netherlands) in both Linköping (at CMIV) and Norrköping. The protocol is efficiently condensed, and the data will be acquired within 50 min.

### Vibration-controlled transient elastography (VCTE)

Transient elastography (TE) relies on a transient mechanical vibration used to induce a shear wave in a tissue. The propagation of the shear wave is then tracked using ultrasound to assess the shear wave speed. A specific implementation of 1D-TE, vibration controlled TE (VCTE), has been developed to assess the average liver stiffness that correlates with liver fibrosis assessed by liver biopsy [33]. In this study, it will be implemented using FibroScan^®^, which is available at both project sites (Linköping and Norrköping), including M- and XL-probe as well as Controlled attenuation parameter (CAP). CAP measures liver ultrasonic attenuation on the signals acquired by the FibroScan^®^. Principles of CAP measurements has been described elsewhere [34].

### Liver biopsy

Patients with increased values of Fibroscan^®^ or MRE will be offered a liver biopsy to confirm the fibrosis stage. Liver biopsies will be performed according to the clinical routine at the University Hospital in Linköping. All biopsies will be performed with ultrasound guidance, using a 1.6-mm Biopince^®^ needle. A pathologist with NAFLD experience will assess the biopsies. The following histopathological variables will be recorded: steatosis, lobular inflammation, hepatocellular ballooning, portal inflammation, Mallory-Denk bodies, and portal/pericellular fibrosis. The pathologist will also perform a global assessment for the presence of NASH [35].

#### 3-Year follow-up

Follow-up will be performed 3 years after the initial examination to assess the progression or regression of incidence of NAFLD and advanced hepatic fibrosis, as well as development of cardiovascular and liver-related events. A complete list of clinically significant events is presented in Table 4. The follow-up protocol is planned to be identical to the baseline protocol, although minor changes may be done after a renewed ethical review due to development within the research field.

**Table 4:** Clinically significant events registered at follow-up after 3 years of follow-up

Event category	
Death	
Cause of death	
Major Cardiovascular Event	
Non-fatal stroke	
Non-fatal myocardial infarction	
Coronary revascularization	
Hospitalisation for heart failure	
Atrial fibrillation	
Hepatic	
Diagnosis of cirrhosis	
Diagnosis of any cirrhosis complication	
Varices or variceal haemorrhages	
Ascites	
Encephalopathy	
Hepatocellular carcinoma	
Liver transplantation	

#### Time plan and implementation

The project involves patient recruitment, data acquisition, and data analysis. Patient recruitment started in March 2019. The goal is to complete patient inclusion in 2022.

In terms of data acquisition, the MR-scanners have been operational since March 2020.

Repeated 3-year MR imaging is expected to be concluded in 2026.

## Discussion

NAFLD is the most prevalent liver disease worldwide and is strongly associated with increased mortality from cardiovascular disease (CVD). NAFLD is rapidly becoming the leading cause of advanced liver disease in Western countries, and is the main reason for liver transplantation [36–38]. Albeit being highly prevalent, only a minority of patients with NAFLD (4–10%, depending on the follow-up time) will progress to cirrhosis and end stage liver disease [39]. However, because of its high heterogeneity, it is challenging to identify NAFLD patient at risk of progression [40]. Although fibrosis stage is a robust predictor of liver related morbidity and all-cause mortality in patient with NAFLD [6], the use of liver biopsy in a routine clinical setting, for a highly prevalent disease, is not realistic. Therefore, noninvasive modalities for the diagnosis of advanced fibrosis have been proposed. Magnetic resonance elastography and vibration controlled transient elastography are two highly validated systems with high negative predictive values but low positive predictive values for the detection of advanced hepatic fibrosis. A low value obtained with MRE or VCTE excludes advanced fibrosis with high precision.

The burden of T2DM is at an all-time high, expected to increase in parallel with the obesity pandemic [11, 41]. The hallmark, *i.e.* insulin resistance, does not only result in development of T2DM, but also NAFLD; therefore, the three conditions are seen as intertwined [42, 43]. Furthermore, T2DM has shown to predict progression to severe liver disease and development of HCC in individuals with NAFLD [44]. This spurred the EASL, EASO and EASD to recommend screening for the presence of hepatic fat and advanced fibrosis in individuals with T2DM [45]. The characteristics of T2DM patients with advanced hepatic fibrosis are unknown, as are the potential mechanisms of advanced hepatic fibrosis in NAFLD and T2DM, and factors that potentiate the development of health complications in T2DM patients with NAFLD.

Moreover, NAFLD is an important cardiovascular risk factor [46, 47]. However, the relationship between NAFLD and myocardial fibrosis and dysfunction in participants with T2DM is poorly understood. One important factor is the dysregulation of altered hormonal gene regulation associated with NAFLD, with increased pro-atherogenic inflammatory markers, procoagulant factors, and disrupted metabolic equilibrium [46]. Hence, the accelerated atherogenesis in individuals with NAFLD probably has its origin in the visceral and hepatic lipid accumulation, with the liver being both the target of the resulting systemic abnormalities and a source of pro-atherogenic molecules that amplify the arterial damage and alter cardiac structure [48, 49]. Furthermore, cardiovascular disease and mortality is prevalent among patients with NAFLD, where the risk of incident CVD and the risk of developing CVD is independently increased [50, 51]. Similarly, the need for coronary angiography, myocardial fibrosis and percutaneous coronary intervention is more common in patients with NAFLD [48, 52, 53]. However, albeit individuals with NAFLD are more prone to a dismal cardiometabolic risk profile, hepatic steatosis is not independently associated with CVD [54, 55]. Therefore, the relationship between NAFLD and CVD is poorly understood, with few predictive markers identified.

Most studies evaluating the outcomes of NAFLD patients have been performed at university hospitals where the referred patients are more likely to have advanced NAFLD and are not necessarily representative of the “general” NAFLD patient population. Therefore, in this prospective study, the *'Evaluating Prevalence and Severity Of NAFLD In Primary care'* (EPSONIP) trial (ClinicalTrials.gov Protocol Record 2018:176-31), we will recruit participants from primary care, where the vast majority of NAFLD and T2DM patients are managed in Sweden. We propose to evaluate the utility of advanced non-invasive imaging approaches and serum biomarkers in assessing advanced hepatic fibrosis, myocardial fibrosis, or myocardial dysfunction in patients with T2DM. We anticipate that development of reliable non-invasive methods to diagnose hepatic and myocardial fibrosis proposed herein will enable timely identification of patients with NAFLD and T2DM, at risk of developing future complications. Identification of these patients will allow early prevention, offering evolving pharmacological therapies, and providing monitoring and treatment of life-threatening complications. Unnecessary follow-up will thus be avoided in patients at low risk of developing future complications. In the long term, this will improve the care and quality of life of the affected individuals, and spare costs to healthcare providers as well.

## Supplementary Information

**Additional file 1.** Supplementary tables.

## Data Availability

The dataset during and/or analysed during the current study available from the corresponding author on reasonable request.

## Abbreviations

AUDIT: Alcohol use disorder identification test
CAP: Controlled attenuation parameter
CMIV: Center for Medical Image Science and Visualization
CVD: Cardiovascular disease
EASD: European Association for the Study of Diabetes
EASL: EAS the liver
EASO: EAS obesity
EPSONIP: Evaluation the Prevalence and Severity of NAFLD in Primary care
GDPR: General data protection regulation
HCC: Hepatocellular carcinoma
ICH-GCP: International Council for Harmonisation of Technical Requirements for Pharmaceuticals for Human Use—Good Clinical Practice
IFIS: International fitness scale
LDH: Lifetime drinking history
MR: Magnetic resonance
MRE: MR elastography
MRI: MR imaging
MRS: Magnetic resonance spectroscopy
NAFLD: Non-alcoholic fatty liver disease
NASH: Non-alcoholic steatohepatitis
PDFF: Proton density fat fraction
T2DM: Type 2 diabetes mellitus
TE: Transient elastography
QoL: Quality of life
VCTE: Vibration controlled TE
