# Supplementary material for: Evaluating the prevalence and severity of NAFLD in primary care: the EPSONIP study protocol
Source: BMC Gastroenterol. 2021 Apr 20;21:180. doi: 10.1186/s12876-021-01763-z (PMC8056630; doi:10.1186/s12876-021-01763-z)
Supplement: Supplementary file 1 — Additional file 1. Supplementary tables. [file 12876_2021_1763_MOESM1_ESM.docx]

**Supplementary table 1.**

|  | Calculation formula |
| --- | --- |
| NAFLD fibrosis score | -1.675 + 0.037 x age (yrs) + 0.094 x BMI (kg/m^2^) +1.13 x impaired fasting glucose/diabetes (yes = 1, no = 0) + 0.99 x AST/ALT ratio – 0.013 x platelet count (x10^9^/L) – 0.66 x albumin (g/dL). |
| APRI | (AST (U/L)/(AST upper limit of normal))/(platelet count (×10^9^/L) × 100) |
| FIB-4 | (age (yrs) x AST (U/L)) / ((platelet count (×10^9^/L)) x (ALT(U/L))^1/2^) |
| BARD | BMI > 28 kg/m^2^ = 1, AST/ALT > 0.8 = 2, presence of diabetes = 1 |
| NIKEI | -24.214 + 0.225 x age (yrs) + 0.056 x AST (U/L) + 5.044 x AST/ALT ratio + 3.631 x bilirubin (mg/dL) |
| NASH-CRN regression score | 0.0539 x body weight (kg) + 10.5166 x glucose (mmol/L) + 10.0695 x AST (U/L)-0.0189 x ALT (U/L) - 0.0594 x prothrombin index (%) -3.6323 |
| King´s score | ((age (years) x AST (U/L) x prothrombin (INR)) / (platelet count (10^9^/L)) |
| GUCI | AST (µkat/L) / (AST upper limit of normal) (µkat/L) x prothrombin (INR) x 100 / platelet count (× 10^9^/L) |
| Lok index | log odds = - 5.56 – 0.0089 x platelet count (10^3^/mm^3^) + 1.26 x (AST/ALT) + 5.27 x INR;  Lok = [exp (log odds)]/[1 + exp (log odds)] |
| Forns score | 7.811 - 3.131 x ln [platelet count (10^9^  /L)] + 0.781 x ln [GGT(U/L)] + 3.467 x ln [age (yrs)] – 0.014 [cholesterol (mg/dL)] |
